# A New Variant of Choledochal Cyst-Type 6B

**DOI:** 10.7759/cureus.16273

**Published:** 2021-07-08

**Authors:** Sugumaran K, Hirdaya H Nag

**Affiliations:** 1 Surgical Gastroenterology, Govind Ballabh Pant Institute of Postgraduate Medical Education and Research, New Delhi, IND

**Keywords:** cdc, choledochal cyst, choledochal cyst type 1d, choledochal cyst type 6b, cystic duct dilatation

## Abstract

A choledochal cyst involving the cystic duct (type 6) is a rare disease. Dilatation of the common bile duct along with the involvement of cystic duct is extremely rare and only a few cases have been reported until now. A 38-year-old woman was evaluated for complaints of pain in the upper abdomen. On initial imaging with ultrasonography (USG) and magnetic resonance cholangiopancreatography (MRCP), she was diagnosed to have a type 1a choledochal cyst. Intraoperatively we found that it was not a simple cystic dilatation of the common bile duct (CBD) alone but the cystic duct was also dilated. Simple cholecystectomy with excision of the cyst and reconstruction with a Roux en Y hepaticojejunostomy was performed. The patient is doing well after six months of follow up. This type of choledochal cyst (CDC) with combined dilatation of cystic duct and CBD has not been defined in the Todani classification. Two studies published until now have given their own extension of the Todani classification as either type 1D or 6B. Our another case where a 23-year-old female with similar complaints was diagnosed with an isolated cystic duct dilatation (type 6A); here, we did a simple laparoscopic cholecystectomy. Thus, we need to know that these distinct type of choledochal cyst exists and has to be added to the classification. It is also important to classify type 6 into two types A and B as their management differs.

## Introduction

Choledochal cysts are either congenital or acquired dilatation of the biliary tree involving either the intrahepatic or extrahepatic biliary radicals or both. Alonso-Lej et al. [1] described three types of choledochal cysts in 1959, which were later modified by Todani et al. [2]. The primary classification of the choledochal cyst (CDC) by Todani et al. includes five variants. The sixth variant of isolated dilatation of the cystic duct was recently described. We present first an extremely rare case of combined dilatation of the common bile duct and cystic duct in a 38-year-old female evaluated for upper abdominal pain in our institution. Preoperatively she was diagnosed to have type 1 choledochal cyst but intraoperatively we found involvement of the cystic duct also. This type of presentation is very rare and few cases have been reported in the literature earlier, but these have not been included in the Todani classification and are not standardized. Some have reported these as a variant of type 6-type 6B involving combined dilatation of cystic duct as well as the common bile duct (CBD) [3]. In another series of six cases where a new variant of choledochal cyst with the participation of the cystic duct has been described and they have proposed the classification of this type of choledochal cyst as a new subtype of Todani I cyst, namely type ID [4]. Thus, there is a need for a clearly defined classification system for these cysts as they may be categorized as either type 1D or type 6B cysts.

Our second case is also a rare case of isolated cystic duct dilatation in a 23-year-old female who presented with pain abdomen and a laparoscopic simple cholecystectomy was done. This type of CDC with cystic duct involvement is usually classified as type 6. But now there are many cases being reported in the literature with combined dilatation of cystic duct and rest of biliary tract. We present these cases to appreciate this type of choledochal cyst as a distinct entity and thus addition to the existing literature, for a standardized classification for these variants. This case has been reported in line with the surgical case report (SCARE) criteria [5].

## Case presentation

**Figure 1:**
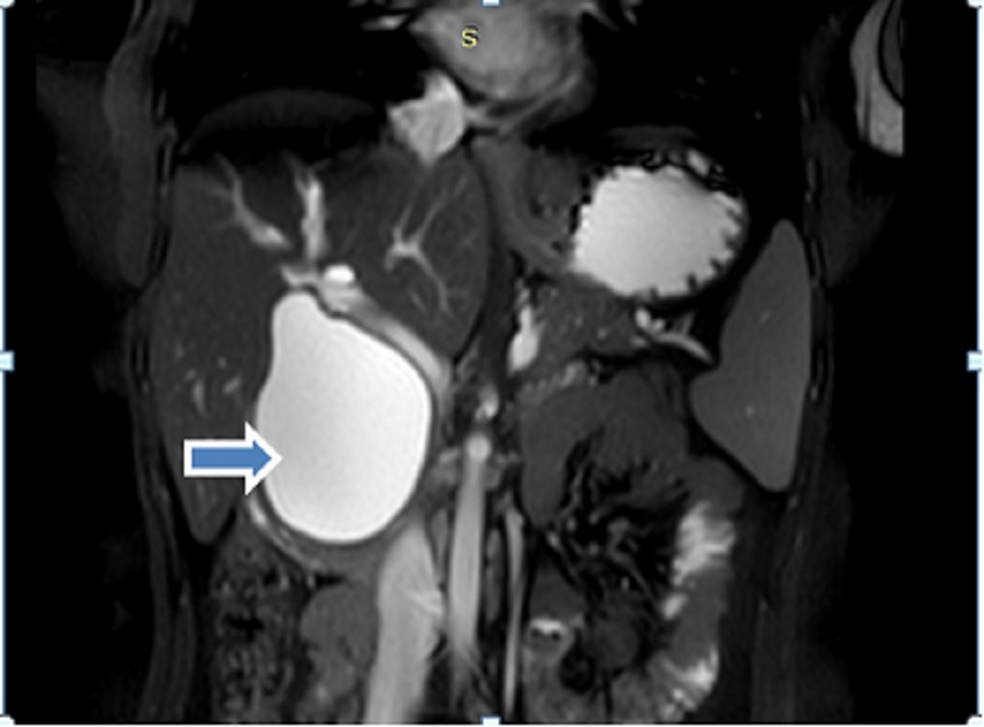
Preoperative MRCP image Axial cut showing hugely dilated CBD (arrow showing dilated CBD). MRCP: magnetic resonance cholangiopancreatography, CBD: common bile duct.

A 38-year-old female presented to our hospital with complaints of pain in the upper abdomen for six months with increased intensity since last one month which was not related to food intake and was not radiating to the back. She had no symptoms of jaundice and fever. On abdominal examination, there was no palpable mass or other significant findings. There were no abnormal findings in the laboratory test. USG abdomen revealed cystic dilation of CBD with the normal gall bladder. Further evaluation with magnetic resonance cholangiopancreatography (MRCP) abdomen revealed dilated cystic structure seen in the region of CBD measuring about 55 mm with smooth distal tapering suggestive of CDC type 1a according to Todani classification (Figure 1).

**Figure 2:**
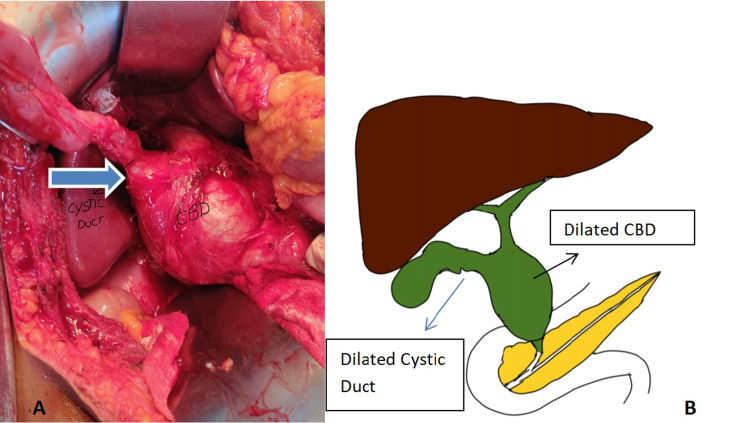
Intraoperative image and its schematic representation (A) Large dilated CBD extending to the proximal cystic duct (indicated by the block arrow); (B) schematic representation of combined dilation of cystic duct and CBD with normal CHD. CBD: common bile duct, CHD: common hepatic duct.

Intraoperatively gall bladder was mildly distended with no calculi. Cystic dilatation of suprapancreatic part of CBD around 6 cm in largest dimension with dilated and tortuous cystic duct of around 2 cm opening into it (Figure 2).

We performed a simple cholecystectomy with choledochal cyst excision with Roux en Y Hepaticojejunostomy. Post-operatively she was started orals on POD 2 and was discharged by POD 5. The post-operative pathology report showed features of choledochal cyst with chronic inflammatory pathology without evidence of malignancy. After a follow-up of six months, the patient is doing well without any morbidity.

**Figure 3:**
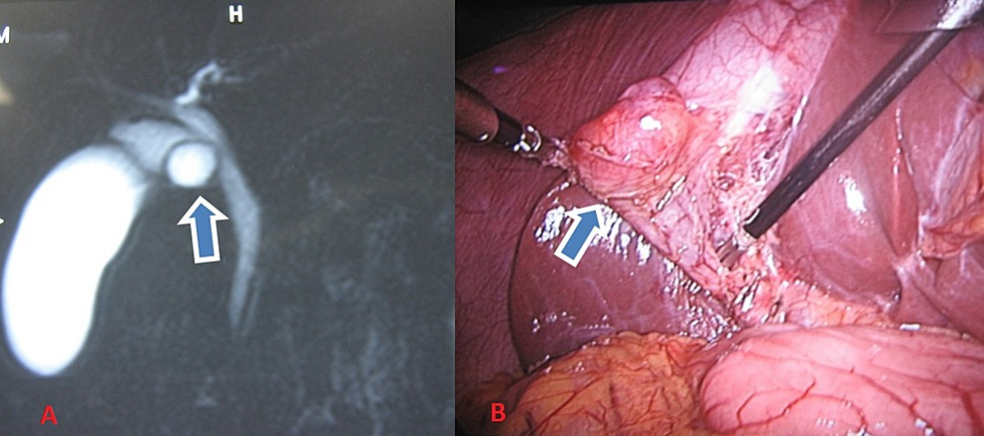
Preoperative and intraoperative images (A) MRCP showing cystic lesion (arrow pointing the cyst) at the neck of gall bladder; (B) intraoperative finding showing isolated cystic duct dilatation (shown by arrow). MRCP: magnetic resonance cholangiopancreatography.

Another case was a 23-year-old female with complaints of only pain in the right upper abdomen for four months, with no symptoms of jaundice and fever. Clinical examination revealed no significant findings. Laboratory investigations revealed no abnormality. On initial evaluation with ultrasonography of the abdomen showed a hypoechoic lesion in the region of GB neck with no gall stones. On further evaluation with MRCP was found to have a cystic lesion at the GB neck with a normal CBD. Preoperatively she was diagnosed as type 2 CDC. Intraoperatively, it was an isolated cyst of the cystic duct (type 6A) of size 2 × 2 cm^2^ with a narrow opening at its juncture with CBD, and CBD was normal (Figure 3). Laparoscopic simple cholecystectomy was done. Post-operative histopathology report showed cyst wall lined by biliary epithelium and peribiliary glands with acute or chronic inflammatory infiltrate without any evidence of malignancy. The post-operative period was uneventful. She is doing well after five years of follow-up.

## Discussion

Choledochal cysts are rare congenital anomalies characterized by cystic dilatations of the intrahepatic and/or extrahepatic bile ducts. The incidence is seen to be increased in the Asian population and especially in Japan [6]. The first classification system for choledochal cysts was given by Alonso-Lej et al. in 1959, describing only three types that were later modified by Todani et al. and most commonly followed. Isolated cystic dilatation of the cystic duct was not included in the Todani classification. Serradel et al. first described a case of isolated dilatation of the cystic duct known as type 6 choledochal cyst a new addition to the Todani classification [7]. After this many such cases of type 6 have been reported in the literature. Most cases of CDC are asymptomatic or present with non-specific upper abdominal pain, palpable mass, obstructive jaundice or acute cholangitis develops in some cases. Our patients had only features of upper abdominal pain. After the initial evaluation, our cases were reported as type 1a CDC and type 2 CDC, respectively, in the MRCP report.

But intraoperatively, we came across an unusual finding where both cystic duct and CBD were largely dilated in the first case and isolated cystic duct dilatation in another case. These types of CDC are very rare and have not been described commonly in the literature before. Yoon in 2011 has described three cases of CDC with involvement of cystic duct; in that two cases had involvement of both the cystic and common bile duct, and proposed that these variant anomalies be included in the classification of the choledochal cyst [8]. Michaelides et al. in 2011 had described a series of three cases with the dilatation of the common hepatic duct, common bile duct and dilatation of the central portion of the cystic duct, giving a bicornal configuration to the cyst. They have proposed the classification of this type of choledochal cyst as a new subtype of the Todani I cyst, namely Todani ID [4]. Bhoil et al. in 2015 has published a case of CDC similar to our first case with combined cystic duct and CBD dilatation [3]. They proposed that type 6 cysts should be further divided into two types: type 6A involving isolated dilatation of the cystic duct (type VI as proposed by Serradel et al.) and type 6B involving combined dilatation of cystic duct as well as the CBD (Table 1).

**Table 1:** Studies published with combined cystic duct and CBD dilatation in the choledochal cyst. CDC: choledochal cyst, CBD: common bile duct, CHD: common hepatic duct.

Study	No of cases	Features of the CDC	Classification given
Yoon [8]	3-with involvement of cystic duct	2 had involvement of both the cystic and CBD	Not given
Michaelides et al. [4]	6	Dilatation of the CHD, CBD and dilatation of the central portion of the cystic duct	Type 1D
Bhoil et al. [3]	1	Combined dilatation of cystic duct as well as the CBD.	Type VI B

So in our first case, there was dilatation of both cystic duct and CBD without the involvement of common hepatic duct similar to the case of Bhoil et al., and thus, we label it as a type 6B variant. And in our second case which had isolated cystic dilatation is classified as type 6A. Management of type 6A is simple cholecystectomy if the opening of the cystic duct into CBD is narrow, whereas complete excision of CBD is required as there is a wide opening with the CBD. So this classification is important as the management of type 6A is simple cholecystectomy and type 6B is complete excision of CBD with hepaticojejunostomy.

## Conclusions

We hereby conclude these variant types of CDC exists and many cases may be underreported because of difficulty in diagnosing with imaging alone and unawareness of these type of cysts. CDC with cystic duct involvement (type 6) may be divided into types 6A and 6B as their management differs.
